# The maintenance of long-term weight loss after semaglutide withdrawal in obese women with PCOS treated with metformin: a 2-year observational study

**DOI:** 10.3389/fendo.2024.1366940

**Published:** 2024-04-11

**Authors:** Mojca Jensterle, Simona Ferjan, Andrej Janez

**Affiliations:** ^1^ Department of Endocrinology, Diabetes and Metabolic Diseases, Division of Internal Medicine, University Medical Centre Ljubljana, Ljubljana, Slovenia; ^2^ Faculty of Medicine, University of Ljubljana, Ljubljana, Slovenia

**Keywords:** metformin, semaglutid, PCOS, obesity, maintenance, weight regain

## Abstract

**Background:**

Withdrawal of semaglutide is frequently followed by weight regain due to compensatory biological changes that prevent the maintenance of long-term weight loss. There are some studies implying that metformin might attenuate weight regain. The weight trajectory after discontinuation of short-term semaglutide treatment in obese women with PCOS who continued metformin treatment has not yet been evaluated.

**Aims:**

We explored changes in body weight, cardiometabolic and endocrine parameters in obese women with PCOS who continued treatment with metformin 2 years after discontinuation of short-term intervention with semaglutide.

**Methods:**

25 women with PCOS and obesity, aged 33.7 ± 5.3 years (mean ± SD), were treated with once-weekly subcutaneous semaglutide 1.0 mg as an adjunct to metformin 2000 mg/day and lifestyle intervention for 16 weeks. At week 16, semaglutide was discontinued. Treatment with metformin 2000 mg/day and promotion of lifestyle intervention were continued during the 2-year follow-up period. Weight change, cardiometabolic, and endocrine parameters were assessed 2 years after semaglutide discontinuation.

**Results:**

During semaglutide treatment phase, weight decreased from 101 (90-106.8) kg to 92 (83.3-100.8) kg. Two years after semaglutide withdrawal, weight was 95 (77-104) kg. The net weight loss 2 years after discontinuation of semaglutide remained significant when compared to baseline (p=0.003). At the end of the study, 21 out of 25 subjects had lower body weight compared to baseline. Improvements in cardiometabolic parameters including decrease in total and LDL cholesterol, fasting glucose, and glucose after OGTT that had been seen during semaglutide-treatment phase reverted towards baseline two years after semaglutide cessation. Free testosterone levels significantly decreased during semaglutide treatment from 6.16 (4.07-9.71) to 4.12 (2.98-6.93) nmol/l, (p= 0.012) and did not significantly deteriorate after semaglutide discontinuation.

**Conclusion:**

Two years after semaglutide withdrawal, women with PCOS who continued with metformin regained about one-third of the semaglutide-induced weight loss. At the end of the follow up, 84% of women had a lower body weight than at baseline.

## 1 Introduction

Weight management and improvement in overall health are fundamental goals in treating obesity. While weight loss is achievable, weight maintenance remains a lifetime challenge (1). A significant contributor to weight regain after weight loss is the reduction in resting energy expenditure following weight loss (2). Additionally, altered levels of satiety hormones and the change in the rate of gastric emptying might play pivotal roles (3). There is some evidence of not only intensified appetite post-weight loss but also of an increased preference for high-calorie foods (4). In addition to altering levels of satiety hormones, weight loss has been associated with changes in several key hypothalamic-pituitary neuroendocrine axes that may promote weight regain after the initial weight loss (5). Recent data show that weight loss-induced variations in cellular stress, extracellular matrix remodeling, inflammatory responses, adipokine secretion and lipolysis seem to be associated with the amount of weight that is regained after successful weight loss (6).

A new generation of anti-obesity medications (AOMs) has revolutionized the way obesity is treated, enabling most patients to lose 10-20% of their body weight. Intervention with the latest AOMs even results in 20% weight loss and is already approaching the effectiveness of bariatric surgery. However, after stopping AOM, weight regains due to the compensatory metabolic adjustments described above (7–10). The trajectory of body weight change after discontinuation of the currently most widely used AOM, the glucagon like peptide-1 receptor agonist (GLP-1 RA) semaglutide, was observed in two studies from the STEP program (9, 10). STEP 4 directly compared the effect of semaglutide continuation versus semaglutide discontinuation on body weight (9). After a run-in 20-week treatment period with semaglutide, enrolled participants were re-randomized to continue treatment with semaglutide for 48 weeks or switch to placebo. Continuation with semaglutide resulted in continued weight loss over the following 48 weeks, resulting in 17.4% of net weight loss from baseline, while those switched to placebo regained 6.9% of body weight after the switch to placebo (9). STEP 1 study extension complements the observations from STEP 4 (10). One year after withdrawal of 68 week intervention with semaglutide, participants regained 2/3 of their prior weight loss with residual benefits in some changes in cardiometabolic variables (10).

Metformin, a biguanide anti-hyperglycemic agent, is the first-line treatment for type 2 diabetes (11). In addition, it has demonstrated effectiveness in prediabetes and in insulin-resistant conditions, including polycystic ovary syndrome (PCOS) (11, 12). The exact mechanism of metformin’s action is still unknown. It reduces liver glucose production, enhances insulin sensitivity in body tissues (11), decreases hyperinsulinemia by reducing insulin resistance and increases the secretion of growth/differentiation factor 15 (GDF15), which suppresses appetite and reduces caloric intake (13–16).

In the Diabetes Prevention Program Outcome Study, modest weight loss with metformin was maintained over a 10-year follow-up period, in contrast to the original lifestyle group from Diabetes Prevention Program, which partly regained weight during follow up period (17, 18). In addition, several trials have demonstrated beneficial effects of metformin in reversing or preventing weight gain associated with antipsychotic drug therapy (14). More specifically, it was more effective in preventing antipsychotic induced weight gain in first episode patients than in chronic patients who have already gained weight implying that it shows more efficacy in preventing weight gain before the onset of significant insulin resistance (19). Some studies also imply that metformin could stabilize natural course of progressive body weight regain in women with PCOS (20–23).

Based on its mode of action and some clinical data (17–23), we hypothesized that metformin may partially prevent weight regain after weight loss induced by semaglutide. To date, the amount of weight regain after semaglutide withdrawal in patients with obesity continuing metformin treatment, including obese women with PCOS, has not yet been evaluated. We aimed to explore changes in body weight, cardiometabolic and endocrine parameters in obese women with PCOS who continued metformin treatment 2 years after semaglutide cessation.

## 2 Materials and methods

### 2.1 Trial design

**Figure 1:**
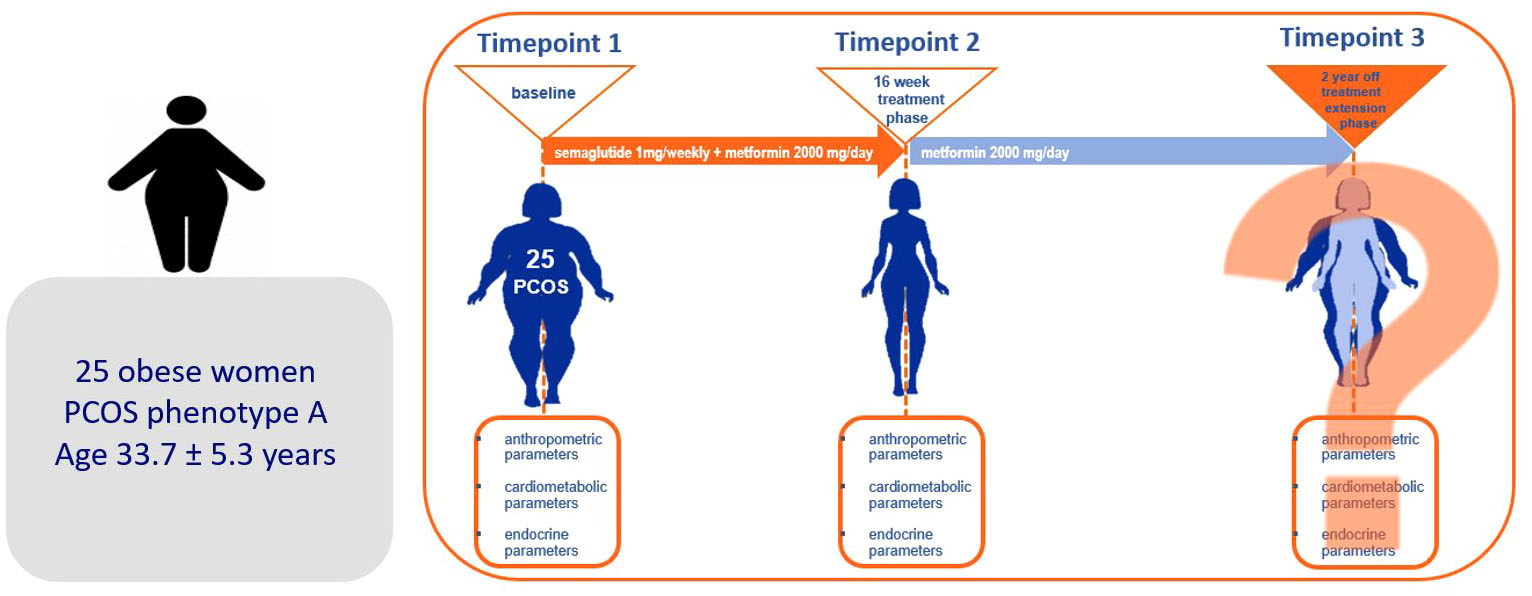
The protocol of the study.

We conducted an observational study including women with PCOS and obesity, followed up at the outpatient clinics of the Department of Endocrinology, Diabetes, and Metabolic Diseases at the University Medical Centre Ljubljana, who continued treatment with metformin 2 years after discontinuation of short-term intervention with semaglutide as an add on to metformin therapy. The trial was conducted in accordance with the Declaration of Helsinki and approved by the National Medical Ethics Committee (approval number 0120-258/2019/12). All subjects were informed of the study’s aims and provided written consent before they were enrolled in the study. The protocol of the study is presented at **Figure 1**.

### 2.2 Trial population

Twenty-five women, aged 33.7 ± 5.3 years (mean ± SD), diagnosed with PCOS and obesity were included in the study. The diagnosis of PCOS was established using the Rotterdam criteria with all included patients characterized as phenotype A. Phenotype A was defined as the concomitant presence of hyperandrogenism, ovulatory dysfunction, and polycystic ovarian morphology (PCOM). At the time of diagnosis, all women were advised to follow healthy lifestyle intervention and to start with metformin treatment up-titrated to 2000 mg/day.

We chose women with PCOS and obesity because weight management in this metabolically compromised population is even more challenging than in the general population. An updated approach with more advanced therapeutic strategies is needed to control weight and cardiometabolic risk factors in the long term. The use of both therapeutic groups, the established metformin and the newer AOMs, in combinational and intermittent regimens seems to be a reasonable option that needs more research attention in this population.

### 2.3 Study protocol

The participants were treated with semaglutide, 1.0 mg/week in addition to their ongoing treatment of metformin at a dosage of 2000 mg/day and lifestyle interventions for 16 weeks. The starting dose of semaglutide was 0.25 mg/week for 2 weeks, followed by 0.5 mg/week for 2 weeks, escalating to 1.0 mg/week for 12 weeks. At the end of week 16, the use of semaglutide was discontinued and the participants underwent regular follow-up outpatient controls to monitor their anthropometric, biochemical and hormonal parameters. Throughout the two-year follow-up period, they continued their treatment with metformin at a dosage of 2000 mg per day, along with promotion of healthy lifestyle intervention.

### 2.4 Assessment of anthropometric and biochemical parameters

At baseline (timepoint 1), at the end of semaglutide intervention (timepoint 2), and at the end of the study (timepoint 3), all participants underwent body weight assessment. A fasting blood sample was drawn for determination of glucose luteinizing hormone (LH), follicle-stimulating hormone (FSH), androstenedione, dehydroepiandrosterone sulphate (DHEAS), total and free testosterone (T), and lipids, followed by a 2-h OGTT. Blood samples for glucose were drawn at 0 and 120 min of OGTT. Safety clinical assessment was performed at the beginning and the end of the treatment with semaglutide period.

We performed a standard 75 g oral glucose tolerance test (OGTT) (24). Glucose levels were determined using a standard glucose oxidase method (Beckman Coulter Glucose Analyzer, Beckman Coulter, Inc., CA, USA). Lipids were determined using Adiva 1800, Siemens analyzer. For measurements of hormone levels, the samples were collected in the morning in a fasting condition. Androstenedione and DHEAS were measured by specific double antibody RIA using 125 I-labeled hormones (Diagnostic Systems Laboratories, Webster, Tx). Total and free testosterone levels were measured by a coated tube RIA (DiaSorin, S. p. A, Salluggia, Italy and Diagnostic Products Corporation, LA, respectively). The intra-assay coefficient of variation (CV) for androstenedione ranges from 5.0 to 7.5% and the inter-assay CV ranges from 4.1 to 11.3%, the intra-assay CV for dehydroepiandrosterone sulfate (DHEAS) ranges from 4.9 to 9.8% and the inter-assay CV ranges from 7.9 to 13.0%. The intra-assay CV for free testosterone is 7.7–19.3%, and the inter-assay CV is 6.4–13.2%. The intra-assay CV for total testosterone is 5.1–16.3%, and the inter-assay CV is 7.2–24.3%.

### 2.5 Statistical analysis

In the statistical analysis, continuous variables were described using median and 25-75% range. Normality of the distribution was evaluated using Shapiro-Wilk test and as several variables were not normally distributed, non-parametric tests were used for statistical analyses. For comparison of continuous variables in different time points, Friedman (comparison of three time points) and Wilcoxon signed-rank test for related samples with post hoc Bonferroni corrections for multiple comparisons (comparison of two time points) were used. Statistical analysis was performed using IBM SPSS Statistics, version 27.0 (IBM Corporation, Armonk, New York, USA).

## 3 Results

Baseline characteristics of the study population are provided in **Table 1**.

**Table 1:** Comparison of clinical parameters at baseline (Timepoint 1) with treatment phase (Timepoint 2) and off treatment phase (Timepoint 3).

	Timepoint			Comparison of all three time points	Comparison between time points 1 and 2	Comparison between time points 1 and 3	Comparison between time points 2 and 3
Characteristic	1	2	3	P^a^	P^b^	P^b^	P^b^
Weight (kg)	101 (90-106.8)	92 (83.3-100.8)	95 (77-104)	<0.001	<0.001	0.003	1.000
BMI (kg/m2)	36.4 (33.2-38.6)	33.5 (29.6-36.4)	34.4 (30.4-36.5)	<0.001	<0.001	0.003	1.000
Fasting plasma glucose (mmol/l)	4.8 (4.5-5.1)	4.4 (4.2-4.8)	4.7 (4.4-4.9)	0.001	0.012	1.000	0.027
Fasting plasma glucose (mg/dL)	86.4 (81.0-91.8)	79.2 (75.6-86.4)	84.6(79.2-88.2)	0.001	0.012	1.000	0.027
Glucose 0 min OGTT (mmol/l)	5.3 (4.9-5.8)	5.1 (5.0-5.4)	5.5 (5.2-5.9)	0.005	0.051	0.198	0.015
Glucose 0 min OGTT (mg/dl)	95.4 (88.2-104.4)	91.8(90.0-97.2)	99.0(93.6-106.2)	0.005	0.051	0.198	0.015
Glucose 120 min OGTT (mmol/l)	6.1 (5.5-6.8)	4.7 (4.4-5.6)	5.8 (4.9-8)	0.001	<0.001	1.000	0.015
Glucose 120 min OGTT (mg/dll)	109.8 (99.0-122.4)	84.6 (79.2-100.8)	104.4 (88.2-144.0)	0.001	<0.001	1.000	0.015
Cholesterol (mmol/l)	4.8 (4.6-5.6)	4.8 (4.1-5.6)	5.1 (4.4-6.2)	<0.001	0.033	1.000	0.006
HDL cholesterol (mmol/l)	1.4 (1.2-1.6)	1.2 (1.1-1.4)	1.5 (1.2-1.9)	0.001	<0.001	1.000	0.009
LDL cholesterol (mmol/l)	2.9 (2.7-3.5)	2.9 (2.3-3.3)	3 (2.5-4.2)	0.002	0.006	1.000	0.024
TG (mmol/l)	1.3 (0.9-2.05)	1.1 (1.0-1.5)	1 (0.8-2.3)	0.077	0.102	1.000	1.000
FSH (E/l)	6.1 (4.6-7.3)	4.7 (3.7-5.1)	4.9 (4-7.6)	0.061	0.138	1.000	1.000
LH (E/l)	6.8 (4.1-8.7)	6.4 (3.5-11.3)	4.7 (3.8-11.3)	0.150	1.000	1.000	0.417
DHEAS (µmol/l)	5.02 (3.83-9.38)	6.16 (4.43-8.61)	5.1 (3.65-8.05)	0.327	0.510	1.000	1.000
SHBG (nmol/l))	28.9 (20.1-41.2)	32.9 (24.7-44.2)	37 (26.0-55.3)	0.034	0.096	0.255	1.000
FAI	3 (2-5.5)	3 (2-4)	3 (2.3-3.5)	0.299	0.018	0.465	1.000
Total testosterone (nmol/l)	0.95 (0.69-1.56)	0.69 (0.69-1.29)	1 (0.8-1.5)	0.274	0.699	0.708	0.195
Calculated free testosterone (pmol/l)	16 (12-30)	14 (10-20)	15.5 (12-20.3)	0.681	0.057	1.000	1.000
Calculated free testosterone (%)	1.89 (1.54-2.23)	1.71 (1.43-2.01)	1.61 (1.29-1.88)	0.043	0.015	0.351	1.000
Calculated bioavailable. testosterone (nmol/l)	0.41 (0.30-0.72)	0.35 (0.26-0.51)	0.37 (0.32-0.48)	0.662	0.123	1.000	1.000
Calculated bioavailable. testosterone (%)	46.2 (38-56.35)	43.9 (36.8-51.3)	40.5 (31.0-46.7)	0.135	0.162	0.306	0.948
Free testosterone (pmol/l)	6.16 (4.07-9.71)	4.12 (2.98-6.93)	5.37 (2.72-6.66)	0.026	0.012	0.147	1.000
Androstendione (nmol/l)	6.62 (4.36-8.77)	5.49 (3.78-6.84)	5.71 (4.85-7.19)	0.087	0.006	0.069	1.000

HDL High-density lipoprotein cholesterol; LDL, Low-density lipoprotein cholesterol TAG, triglycerides; FSH, follicle stimulating hormone; LH, luteinizing hormone; DHEAS, dehydroepiandrosterone sulphate; SHBG, sex hormone-binding globulin; FAI, free androgen index; OGTT, oral glucose tolerance test.

Data are presented as median (25%-75%).

^a^compared with Friedman test for related samples; ^b^compared with Wilcoxon signed-rank test for related sample with post hoc Bonferroni correction.

### 3.1 Changes in anthropometric measurements

**Figure 2:**
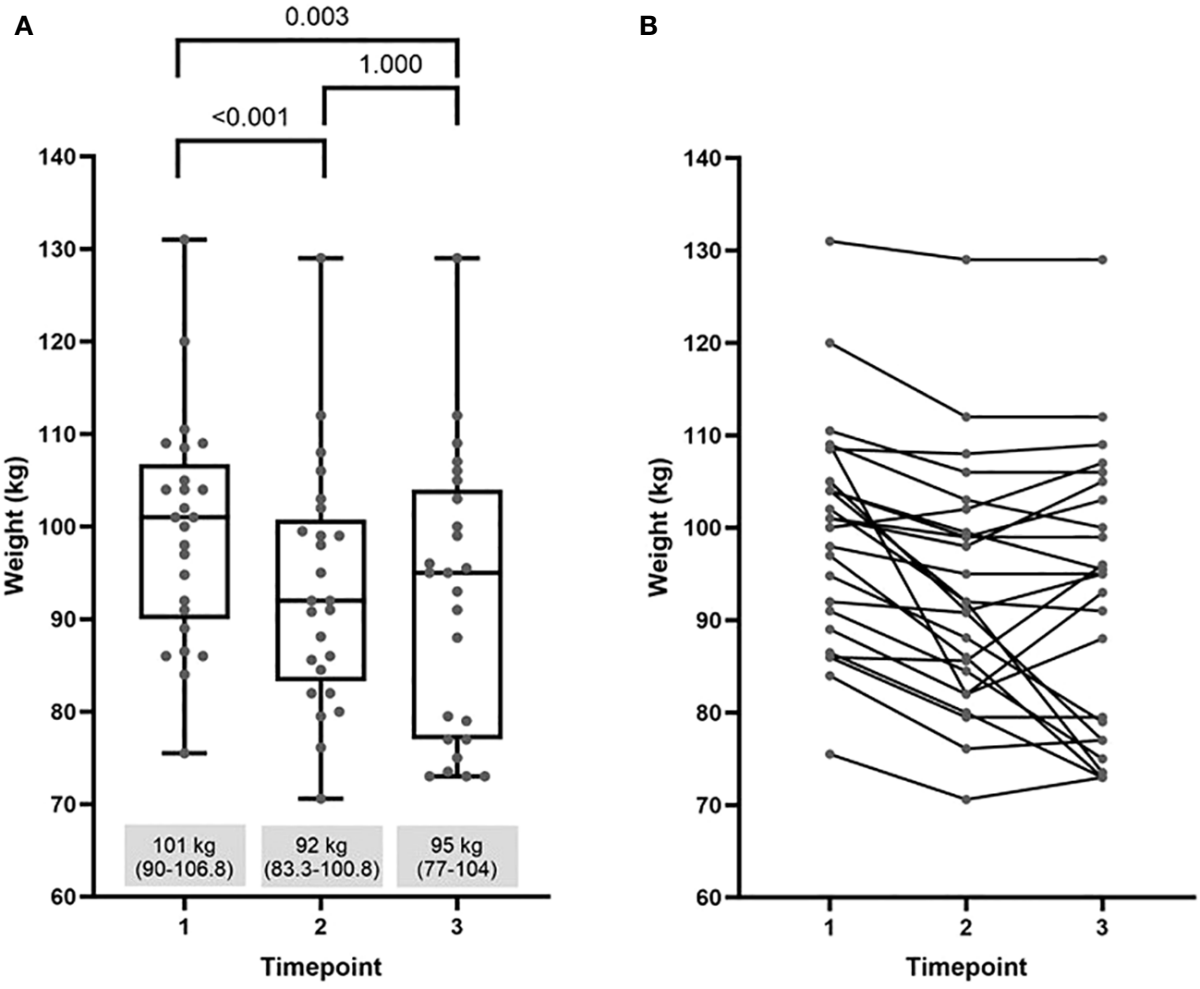
Comparison of weight at baseline (timepoint 1) with values at the end of semaglutide intervention (timepoint 2) and at the end of the study (timepoint 3): **(A)** median (25%-75%) and **(B)** individual weight trajectories.

Overall, during semaglutide treatment phase, from timepoint 1 to timepoint 2, women lost a significant amount of body weight. After discontinuation of semaglutide, they regained about one third of prior weight loss, still resulting in statistically significant net weight loss from the beginning to 2 years after semaglutide cessation. Data are presented in **Figure 2A**. The weight trajectories of individuals are graphed in **Figure 2B**.

### 3.2 Cardiometabolic parameters

**Figure 3:**
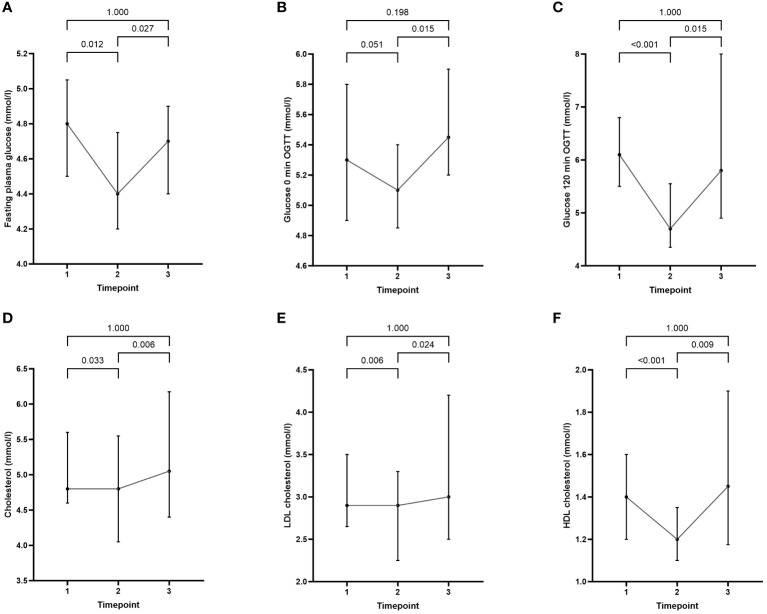
Comparison of cardiometabolic parameters at baseline (timepoint 1) with values at the end of semaglutide intervention (timepoint 2) and at the end of the study (timepoint 3): **(A)** fasting plasma glucose, **(B)** glucose 0 min OGTT, **(C)** glucose 120 min OGTT, **(D)** cholesterol, **(E)** LDL cholesterol, and **(F)** HDL cholesterol. Data are presented as median (25%-75%).

Two years after semaglutide cessation, all observed metabolic parameters reverted toward baseline (**Table 1**). Metabolic improvements during semaglutide treatment phase from timepoint 1 to timepoint 2 included a statistically significant decrease in total and LDL cholesterol, fasting plasma glucose, and glucose on 120 min of OGTT. Contrary to other beneficial changes, HDL cholesterol has been reduced during semaglutide treatment. Data are presented in **Figure 3**.

According to the OGTT at the beginning of the study (timepoint 1), 21 women had normal glucose tolerance (NGT), 3 had impaired fasting glucose (IFG), and 1 had impaired glucose tolerance (IGT). At the end of semaglutide therapy (on timepoint 2), all women had normal glucose tolerance. Two years after semaglutide cessation, 18 women had normal glucose tolerance, 3 had impaired fasting glucose, 3 had impaired glucose tolerance, and 1 developed type 2 diabetes.

### 3.3 Endocrine parameters

The improvement in endocrine parameters induced by semaglutide resulted in the reduction of free testosterone (**Table 1**).

### 3.4 Adverse events

Three subjects experienced intermittent diarrhea, bloating and nausea before the initiation of semaglutide treatment (timepoint 1), but the symptoms were not severe enough to warrant discontinuation of metformin therapy. After starting semaglutide treatment, 11 subjects reported experiencing nausea and dyspepsia, with 3 of them also suffering from constipation. Patients reported that side-effects persisted during the semaglutide dose escalation, after which the symptoms subsided in all, but were still present at the time of discontinuation of semaglutide (timepoint 2). None of the participants discontinued treatment due to gastrointestinal events or other reasons. Two years after discontinuation of semaglutide (timepoint 3), 2 subjects reported occasional liquid stools, 2 experienced occasional nausea and dyspepsia and 1 had occasional bloating. Despite these issues, all participants continued with metformin therapy.

## 4 Discussion

Two years after semaglutide withdrawal, women with PCOS and obesity that continued with metformin treatment regained one-third of their semaglutide-induced weight loss. Improvements of cardiometabolic variables achieved during semaglutide treatment phase reverted to baseline, whereas the reduced free testosterone levels observed during semaglutide treatment did not significantly deteriorate after semaglutide discontinuation.

Women with PCOS face a 2.8-fold higher risk for obesity and a 1.7-fold higher risk for central obesity compared with individuals without PCOS (25). Insulin resistance (IR) occurs early in the course of PCOS and is frequently present even in the absence of obesity, accounting for 60-80% of women with PCOS (26). Metformin in addition to lifestyle intervention has been used in PCOS over the past few decades (27), and should be considered irrespective of glucose homeostasis in all women with PCOS with BMI ≥ 25 kg/m2, because of its expected beneficial effects on metabolic outcomes (28).

AOMs, including liraglutide and semaglutide, combined with lifestyle intervention could offer an upgraded treatment strategy for patients with PCOS and obesity (28). There is growing body of evidence that GLP-1 RAs demonstrate numerous metabolic benefits in PCOS, including a high effect on prediabetes remission rate, a significant reduction in atherothrombotic markers, and an improvement in lipid profile (29–32). The effectiveness of low dose subcutaneous semaglutide in addressing obesity-related complications in PCOS was demonstrated in a study where they investigated the efficacy of a low dose (0.5mg once weekly) of subcutaneous semaglutide in obese PCOS patients who were unresponsive to a lifestyle modification program. After 3 months, almost 80% of the obese PCOS patients in the study achieved a minimum 5% reduction in body weight. In addition, basal insulin levels decreased, and insulin resistance (HOMA-IR) improved. Short-term low dose semaglutide treatment also resulted in normalization of fasting blood glucose in 80% of PCOS women with impaired fasting glucose (33).

Even though GLP-1 RAs seem to have a great potential to improve obesity-related metabolic and reproductive complications in PCOS, their use is currently not widely acknowledged and accepted in this population. The most recent international guidelines recommend that when discussing the use of GLP-1 RA with women with PCOS, the potential side effects and the need for long-term use in weight management should always be considered in the shared decision making with the patient. The authors claimed that this is particularly important given the lack of data on long-term safety (28). Until today, the longest observational period for semaglutide treatment for obesity is 2 years (STEP 5). The treatment led to substantial, sustained weight loss versus placebo and maintained improvements in cardiometabolic parameters. Treatment over 2 years was generally well tolerated with no new safety signals (34).

Contradictory to clinical research data confirming benefits of long-term treatment, semaglutide is frequently used for a significantly shorter period of time in clinical practice. A recently published survey conducted among Israeli endocrinologists, internal medicine physicians and family physicians who practice obesity medicine reported that fewer than 50% of physicians discussed treatment duration with their patients, and 52% of patients discontinued therapy in the first 3 months. Among the main reasons for discontinuation were price of the drug and fear of long-term side effects. The authors commented that the observed clinical practice is predominantly the consequence of the off-label regime and the current shortages of the medication (35). The poor adherence to long term treatment with AOMs evident in clinical practices, requires the design of effective algorithms based on the pathophysiology of obesity, to provide the most effective long-term strategies for sequential treatment with different AOMs and other modalities for body weight maintenance.

There are some data that metformin can attenuate weight regain after weight loss and prolong the maintenance of a stable weight after weight loss in PCOS. A 10-year longitudinal follow-up study conducted at our research center on a cohort consisting of 159 patients with PCOS exhibited that long-term metformin treatment in overweight-obese women with PCOS led to a reduction and stabilization of body weight (22). Furthermore, also in pregnant women with PCOS, administration of metformin during pregnancy resulted in less weight gain during pregnancy and a lesser degree of weight loss in the first year postpartum compared to those who received placebo (20). These findings indicate the potential of metformin therapy in stabilizing weight fluctuation.

The weight trajectory after discontinuation of short-term semaglutide treatment in obese women with PCOS who continued metformin treatment has not yet been evaluated, so we can only provide a comparison with the general population from the STEP 1 extension study. In the STEP 1 study, participants regained two-thirds of their previous weight loss one year after discontinuing semaglutide therapy. In contrast, participants in our cohort only regained about one-third of their prior weight loss during the two-year period after discontinuing semaglutide treatment. Notably, we observed highly heterogeneous responses of the individual subjects, both, while on treatment with semaglutide and in the post-treatment period. At the end of the study, 21 out of 25 subjects had lower body weight compared to baseline. Nine of these subjects continued to lose weight after stopping semaglutide. In 6 subjects, body weight remained stable after discontinuation of semaglutide. Initial support with semaglutide was reported as very transformative for lifestyle changes by some of those individuals. Six subjects started to regain weight after stopping semaglutide, but after 2 years their weight was still lower than at baseline.

Reduced values of free testosterone levels observed during semaglutide treatment did not significantly deteriorate after semaglutide discontinuation during continued treatment with metformin. There is some evidence to suggest that metformin may have some effects on various endocrine parameters, including testosterone levels. Research has indicated that metformin treatment may lead to improvements in hormonal imbalances commonly associated with conditions such as PCOS by enhancing insulin sensitivity and indirectly decreasing biochemical hyperandrogenism. Studies have shown that metformin is superior to placebo in lowering testosterone levels compared to placebo or lifestyle interventions as well as lowering Free Androgen Index (FAI). The ability of metformin to reduce androgen levels in women with hyperandrogenic PCOS is more pronounced in non-obese women (36, 37). A 10-year longitudinal follow-up of a retrospective cohort comprising 159 patients with PCOS treated with metformin reported that the total testosterone and androstenedione decreased to 15.4 ± 47.9% and 11.3 ± 46.4% within first year, with further decrease in total testosterone and androstenedione to 37.8 ± 61.8 and 24.8 ± 40.5% at the fifth year of the follow-up (22). In men, research suggests that metformin treatment may lead to a decrease in serum testosterone levels independent of blood glucose control. In men with type 2 diabetes, metformin therapy can reduce testosterone levels and counteract the testosterone elevation that may accompany improvements in blood glucose levels (38, 39).

The observed changes in total and LDL cholesterol during semaglutide treatment in our cohort were in line with a systematic review and meta-analysis addressing the efficacy and safety of GLP-1 RAs on body weight and cardiometabolic parameters in individuals with obesity and without diabetes and with a systematic review and meta-analysis of semaglutide 2.4 mg including the 2-year STEP 5 trial (40, 41). In contrast to both meta-analyses, we observed a reduction in HDL cholesterol, similar to another study in which 30 obese subjects received subcutaneous semaglutide once weekly at a dose of 1.0 mg or placebo (42). Given the higher power of the results of the meta-analyses, the reduction in HDL-cholesterol in our study was most likely a random result due to the small number of subjects. The cardiometabolic improvements seen during semaglutide treatment reverted towards the baseline in our study, similar to the findings in the STEP 1 study (10).

When interpreting the endocrine and cardiometabolic outcomes, it is important to highlight that participants in our cohort were already treated with metformin before the semaglutide intervention, therefore prior the baseline assessment. Knowing that metformin has impact on these parameters, it is possible that metformin “maxed out” with the observations for those two groups of outcomes.

Regarding safety profile, we observed mild gastrointestinal AE in 44% of participants when semaglutide was added as an adjunct to metformin 2000 mg/day. The observations are in line with a post hoc analysis of gastrointestinal (GI) adverse effects in participants who received concomitant metformin with semaglutide 1mg per week in the SUSTAIN 6 study. This analysis demonstrated that GI side effects occurred in 37.6% of participants receiving semaglutide plus metformin, compared to 40.3% of those receiving semaglutide alone. In the control (placebo) group, side effects occurred in 18% of participants receiving metformin and 19.2% without metformin. Concomitant metformin use was therefore not associated with an increased risk of serious GI adverse events, nausea, vomiting, or discontinuation of the study product throughout the trial in SUSTAIN (43).

The results of our study are subject to several limitations. The main limitation of the present study is the small sample size, which was determined by a small group of patients who had previously received semaglutide and long term follow up protocol. Another limitation of our study is the absence of a control group. However, the principal strength of this study was the long-term observation, providing the first insights into a potential novel strategy for partially overcoming adaptive mechanisms following the cessation of semaglutide-induced weight loss in insulin-resistant populations.

## 5 Conclusion

In conclusion, the role of metformin in attenuation of weight regain after semaglutide discontinuation needs to be explored in randomized controlled studies in different insulin resistant populations. The diverse approaches regarding the use of semaglutide for weight reduction highlight the necessity to guide physicians and standardize the long-term treatment regimen in anti-obesity medicine. Emerging algorithms based on the pathophysiology of obesity should provide the most effective sequencing of AOM and emphasize the importance of a dynamic interplay of different modalities combined with lifelong lifestyle intervention. The diverse individual responses suggest the need for more accurate phenotyping of obesity and PCOS and tailored individual approaches in the future.

## Data availability statement

The original contributions presented in the study are included in the article/supplementary material. Further inquiries can be directed to the corresponding author.

## Ethics statement

The studies involving humans were approved by Slovenian National Medical Ethics Committee (approval number 0120-258/2019/12). The studies were conducted in accordance with the local legislation and institutional requirements. The participants provided their written informed consent to participate in this study.

## Author contributions

MJ: Conceptualization, Data curation, Writing – original draft, Writing – review & editing, Methodology. SF: Data curation, Writing – original draft, Writing – review & editing, Formal Analysis, Investigation. AJ: Conceptualization, Data curation, Supervision, Writing – original draft, Writing – review & editing.
